# Total Number of Lymph Nodes in Neck Dissection and Its Relation to Cancer-Positive Lymph Nodes as a Prognostic Indicator in Aerodigestive Tract Cancers: A Multi-Center Study

**DOI:** 10.7759/cureus.47347

**Published:** 2023-10-19

**Authors:** Mohammed A Nujoom, Hani Z Marzouki, Rawan T Arif, Bushra A Alharbi, Hadi Afandi Al-Hakami, Mohammed Garni, Rolina Al-Wassia, Marwan Al-Hajeili, Mazin Merdad

**Affiliations:** 1 Otolaryngology - Head and Neck Surgery, King Abdulaziz University Faculty of Medicine, Jeddah, SAU; 2 Otolaryngology - Head and Neck Surgery, King Saud bin Abdulaziz University for Health Sciences, King Abdullah International Medical Research Center, Ministry of National Guard Health Affairs, Jeddah, SAU; 3 Otolaryngology - Head and Neck Surgery, King Saud bin Abdulaziz University for Health Sciences College of Medicine, Jeddah, SAU; 4 Radiation Oncology, King Abdulaziz University Hospital, Jeddah, SAU; 5 Oncology, King Abdulaziz University Faculty of Medicine, Jeddah, SAU

**Keywords:** aerodigestive, dissection, neck, ratio, node, lymph

## Abstract

Objectives

Few studies have been conducted on the total number of lymph nodes (LNs) in neck dissection and the lymph node ratio (LNR; number of positive lymph nodes divided by number of excised lymph nodes), or their potential use as a prognostic indicator for cancers of the upper aerodigestive tract (UADT) and its treatment. We aimed to measure the number of lymph nodes dissected and the LNR to assess their prognostic value for cancers of the UADT, as well as their effect on overall survival and disease-free survival.

Methods

We performed a retrospective study of patients diagnosed with cancer of the UADT who underwent neck dissection as the primary or secondary modality of their treatment plan at King Abdulaziz University Hospital and the National Guard Hospital, Jeddah, Saudi Arabia. Data were collected through medical records and analyzed to assess prognosis and calculate survival rates in relation to the number of lymph nodes and LNR.

Results

A total of 121 patients were included: 14 women (11.57%) and 107 men (88.43%). The median age was 60 years and the mean follow-up period was 2.7 years. Of the malignancies, 44.63% were of the oral tongue and 35.54% were laryngeal. A median of 38 lymph nodes were dissected during neck dissections. The distribution of the individual LNRs was characterized by mean values. A mean LNR of 0.04 was considered the cutoff value, an LNR of > 0.04 a high LNR, and an LNR of < 0.04 a low LNR. Kaplan-Meier survival estimates for the cohort showed a three-year overall survival rate of 88% (95% confidence interval [CI]: 77% to 94%) for patients with a low LNR, but 71% (95% CI: 47% to 85%) for patients with a high LNR, which was statistically significant. A similar significant decreasing trend persisted at the four-year follow-up, where the disease-free survival rate was 73% (95% CI: 61% to 82%) for patients with a low LNR compared with 56% (95% CI: 35% to 72%) for patients with a high LNR.

Conclusion

The number of excised lymph nodes in neck dissections and the LNR might be a good prognostic indicator for overall survival and disease-free survival in patients with cancers of the UADT and may serve as a valuable tool in deciding on different treatment plans.

## Introduction

Head and neck malignancies that originate from the oral cavity, oropharynx, nasopharynx, hypopharynx, and larynx are described as cancers of the upper aerodigestive tract (UADT) according to the American Joint Committee on Cancer staging manual [1,2]. A total of 644,000 new cases of UADT cancers are diagnosed annually, most from developing countries. Cancers of the UADT are the sixth most common cancer in Asian countries. In the United States, 3.2% of all newly diagnosed cancers are those of the UADT and they represent 2.2% of all cancer deaths annually [3,4]. The prevalence of cancers of the UADT is considered high in Middle Eastern countries [5], in particular in Saudi Arabia [6]. This high prevalence can be attributed to the higher rates of risk factors, including smoking, tobacco chewing, and the use of a chewable plant in the southern regions of the Kingdom of Saudi Arabia, such as Qat, as well as Shamma and other toxic materials.

Treatment options for cancers of the UADT vary depending on site, stage, nodal status, and other factors. The surgical option for these cancers almost always includes a wide surgical resection of the primary tumor, as well as an appropriate neck dissection, depending on the specific case [7].

Nonetheless, questions about the surgical approach to neck dissection for cancers of the UADT remain unanswered: 1. How should an appropriate neck dissection be measured? 2. What is the basis for an appropriate neck dissection? 3. How many lymph nodes should be dissected? 4. Is there any relation between the number of positive lymph nodes and the patient’s prognosis? 

Although many factors have been linked to an appropriate surgical resection, to date no measurements of these factors have been outlined in the literature.

Lymph node ratio

The lymph node ratio (LNR) is defined as the number of positive lymph nodes excised in a neck dissection divided by the total number of lymph nodes extracted. From a mathematical point of view, the lower the number of positive lymph nodes and the higher the number of total lymph nodes, the lower the LNR.

Two well-conducted studies in Germany [8] investigated the usefulness of LNR as a prognostic indicator for survival. According to the results reported from one of these studies, the optimal LNR predictive of survival in oral squamous cell carcinoma (SCC) was expected to be in the range of 0.09-0.1. The other study was a retrospective analysis of over 30 years (1980-2010) that evaluated the utility of the LNR as a potential prognostic predictor in patients with laryngeal SCC [8]. The authors concluded that patients with an LNR of ≥ 0.09 had a higher hazard ratio of 2.065 for disease-specific survival (DSS) compared with those with a lower LNR (< 0.09). The optimal LNR predictive of survival in laryngeal SCC was expected to be in the range of 0.08-0.1 [8].

In a well-conducted meta-analysis performed in Hong Kong, data were categorized into Group A (with pathological nodal disease) and Group B (with and without pathological nodal disease). A high LNR was significantly related to shorter overall survival (OS) and disease-free survival (DFS) and all cutoff values were shown to be significant. This finding highlights the fact that LNR can be a very good prognostic indicator in oral SCC, suggesting its consideration in future staging systems [9].

In this study, we aimed to examine the LNR as a prognostic indicator in patients with UADT cancers for possible use in future staging systems, as well as to improve the quality of neck dissections by reducing the LNR.

This study was previously presented as a poster presentation at the American Society for Radiation Oncology (ASTRO) on February 24, 2022, in Phoenix, Arizona, USA.

## Materials and methods

The study protocol was reviewed and approved by the institutional review board committee at King Abdulaziz University Hospital (KAUH) and King Abdulaziz Medical City National Guard Hospital, in Jeddah, Saudi Arabia. A retrospective cohort study was designed to investigate the number of lymph nodes in neck dissection and its relation to positive lymph nodes as a prognostic indicator in cancers of the UADT.

Patients and setting

All patients with SCC of the UADT who were diagnosed, treated, and followed at King Abdulaziz University Hospital and King Abdulaziz Medical City National Guard Hospital from 2009 to 2019 were included. All patients were included who underwent surgical resection of the primary tumor, along with neck dissection with or without radiation therapy and with or without chemotherapy. A chart review of these patients was conducted to determine the following parameters: age, gender, risk factors (tobacco and alcohol), site of lesion, TNM staging, treatment protocol, treatment response, and outcome. The medical records of all included patients were reviewed and the data obtained for analysis.

Medical record processing

A team of experienced data collectors initialized the collection steps from the medical records. A data extraction sheet was prepared, which included the demographic data and disease parameters of all patients, including diagnosis, date of diagnosis, site of primary lesion, type of management initiated (surgery or neoadjuvant therapy), type of surgery, level of neck dissection, and so forth. The data collectors were twice given a precise and thorough demonstration of the hospital IT system to ensure that data collection was adequate and to minimize errors. Collection took place from May 2017 to October 2017; the principal investigator supervised the data collectors at every step of the process. All information obtained during the study was kept confidential. An identification number was assigned to each subject to ensure that personal identity remained confidential. Should the information from this study be used in presentations or publications, personal information will be kept strictly confidential.

Statistical analysis

Descriptive statistical analysis was performed to compare the socioeconomic, clinical, and treatment characteristics of the patients. These characteristics included whether the patient started with surgery as the primary treatment or with neoadjuvant radiation therapy with or without chemotherapy, the type of neck dissection performed, the number of lymph nodes and positive nodes extracted, and the LNR; this information was then used to analyze the patients’ prognosis and survival, as well as their overall status. 

Univariate and multivariate statistical analyses were performed to identify whether the total number of lymph nodes or positive lymph nodes or the LNR were significant predictors of outcome in the cohort, over and above the prognostic information already used. Kaplan-Meier curves and log-rank tests were used to assess the significance of the total number of lymph nodes and positive nodes and LNR for OS, DFS, mean survival, and one-year survival rates in relation to age, sex, stage, smoking, and drinking. The Cox proportional hazards model was used to assess the incremental prognostic and predictive values of the total number of lymph nodes and positive nodes and the LNR over and above the other standard and potential prognostic features of the tumor and the patient (age, smoking status, and disease stage). DFS was analyzed according to the patterns of failure in relation to the total number of lymph nodes and positive nodes and LNR. Patterns of failure could include local, regional, and distant metastasis and local, regional, and distant metastatic spread. 

## Results

Descriptive analysis

A total of 121 patients were included: 14 women (11.57%) and 107 men (88.43%). The patients’ median age was 60 years (range 25-95), and the mean follow-up period was 2.7 years. Almost 56% of the sample were from Jeddah. The majority had never smoked (76%) and only 5% were current smokers.

The tumors were classified in accordance with the TNM staging system, the majority being stage IV (44%). See Table 1 for the full distribution.

**Table 1:** Tumor Distribution by Stage

Stage	Frequency	Percentage	Cumulative Percentage
I	25	21.01	21.01
II	25	21.01	42.02
III	17	14.29	56.30
IV	52	43.70	100
Total	191	100	

According to histopathological analysis, moderately differentiated SCC was the most frequently reported type (63%), whereas poorly differentiated SCC was the least reported (~11%). Refer to Table 2 for the full distribution.

**Table 2:** Tumor Distribution by Differentiation of Squamous Cell Carcinoma (SCC)

SCC Differentiation	Frequency	Percentage
Poorly differentiated	12	10.26
Moderately differentiated	74	63.25
Well-differentiated	31	26.50
Total	117	100

In our cohort, 44.63% of patients had oral tongue lesions, 35.54% laryngeal cancer, and 15.7% cancer of the oral cavity. Two patients with nasopharyngeal carcinoma underwent salvage neck dissection; among the remaining patients, one patient had cancer of the floor of the mouth, one of the base of the tongue, and one of the hypopharynx. Table 3 shows the complete distribution.

**Table 3:** Number of Cases of Upper Aerodigestive Tract (ADT) Cancer by Tumor Site

ADT Subsite	Frequency	Cumulative Percentage
Base of tongue	1	0.83
Oral tongue	54	44.63
Floor of mouth	1	0.83
Oral cavity	19	15.7
Larynx	43	35.54
Hypopharynx	1	0.83
Nasopharynx	2	1.65
Total	121	100

Regarding treatment modalities, 40% of patients were treated with surgery followed by adjuvant radiotherapy, 38% had surgery alone as the primary treatment, and 22% had surgery followed by adjuvant chemoradiotherapy. Most patients had no perineural (55%) or lymphovascular invasion (79%). The most commonly used chemotherapy was cisplatin in 67% of patients. Only 31% of the patients had a recurrence of cancer after treatment, and 27% of patients were lost to follow-up. Among those who were followed up, 12% died.

Lymph node ratio

**Figure 1:**
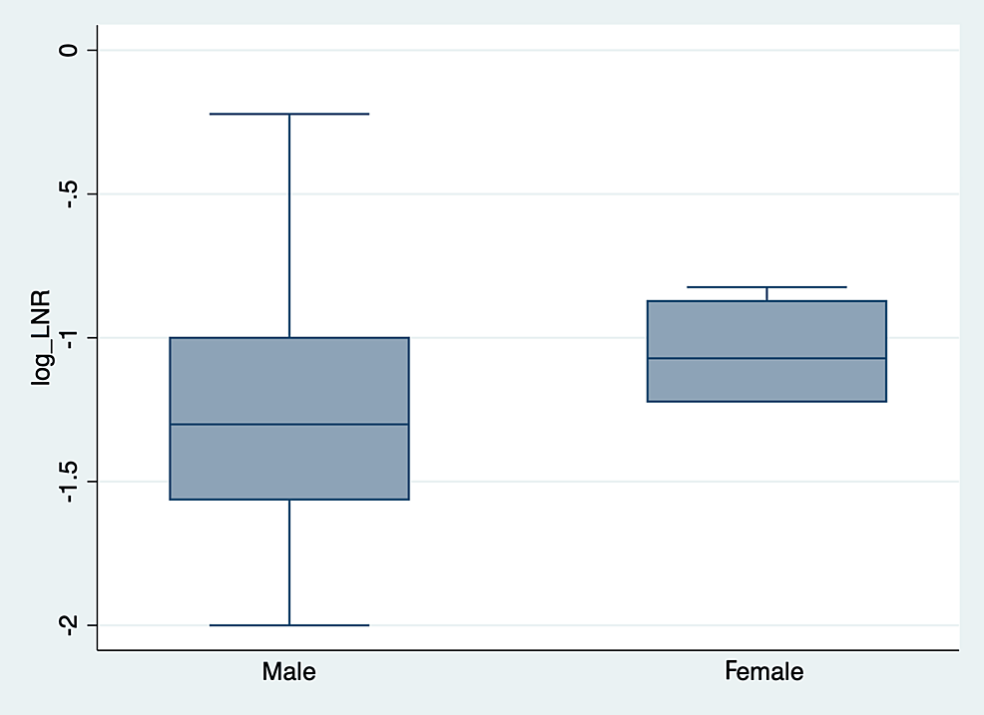
Box Plot Showing the Distribution of the log10 Lymph Node Ratio (LNR) Between Genders

Evaluation of the histopathological reports showed that a median of 38 lymph nodes (mean 43, range 2-228) were dissected during surgery. On reviewing the literature, we found no reference values available for the LNR of head and neck cancer; we therefore decided to characterize the distribution of individual LNRs in our patient cohort according to the mean value (= 0.04). Any LNR above 0.04 was considered to be a high LNR and any results below 0.04 a low LNR. From our observations, women had a lower LNR than men, but this was not statistically significant (Figure 1).

**Figure 2:**
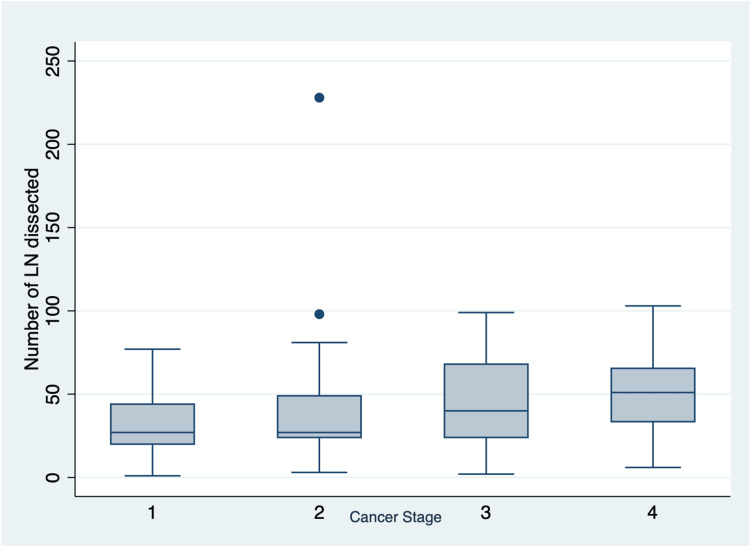
Box Plot Showing the Number of Lymph Nodes (LN) Increasing With Staging

Using regression analysis, we found that the expected average change in the number of lymph nodes dissected between women and men was -3.5, which was not statistically significant (95% CI: 13.3 to -20.4, P = 0.681). Moreover, the expected average change in the number of lymph nodes dissected decreased by -0.34 with each year of increasing age, which was again not statistically significant (95% CI: .4 to -.8, P = .07). The total number of lymph nodes dissected differed by staging, with the larger number of lymph nodes being dissected at stage IV; however, analysis of variance testing showed no statistical significance (Figure 2). 

Overall survival

**Figure 3:**
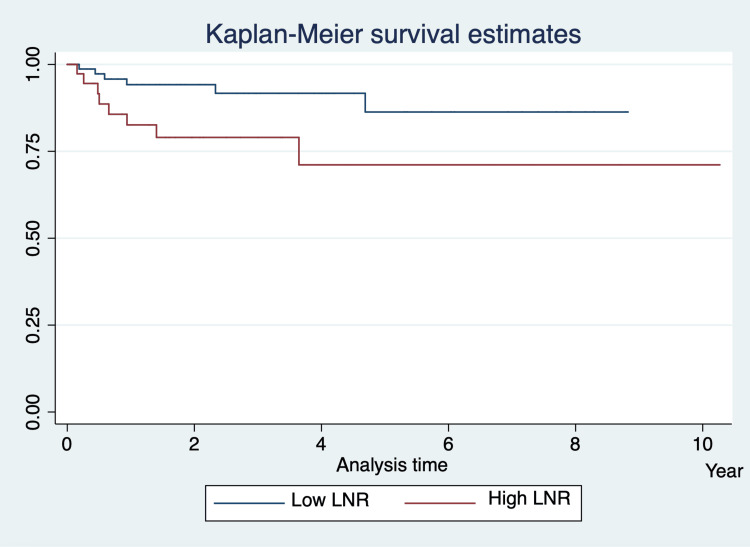
Kaplan–Meier Survival Estimates Showing Three-Year Overall Survival LNR: lymph node ratio

OS was measured from the date of initial diagnosis to the death of the patient, to the time the patient was lost to follow-up, or to the end of the study. According to the Kaplan-Meier survival estimates for the entire cohort, the three-year OS was 88% (95% CI: 77% to 94%) for those with a lower LNR and 71% (95% CI: 47% to 85%) for those with a higher LNR, which was statistically significant (Figure 3).

Differences in proportion of deaths

After the assumption of normality of the data was tested, the null hypothesis of an equal proportion of deaths between LNR groups was tested by using the two-sample test of proportion and it was rejected (P < .05). Those with a higher LNR had a higher proportion of deaths than did those with a lower LNR, with a difference of 0.12 when the former was compared the latter (0.011, - 0.26).

Disease-free survival 

DFS was calculated from the time (in years) of initial diagnosis to the first recurrence, or to the censoring time of the patients (last admission, or death, or did not develop the outcome by the end of the study). According to the Kaplan-Meier survival estimates for the entire cohort, assuming a 0.05 level of significance and using the log-rank test for equality of survivor functions, we concluded that the proportions of recurrence were not the same across the hypothesized LNR categories (P = .000). 

**Figure 4:**
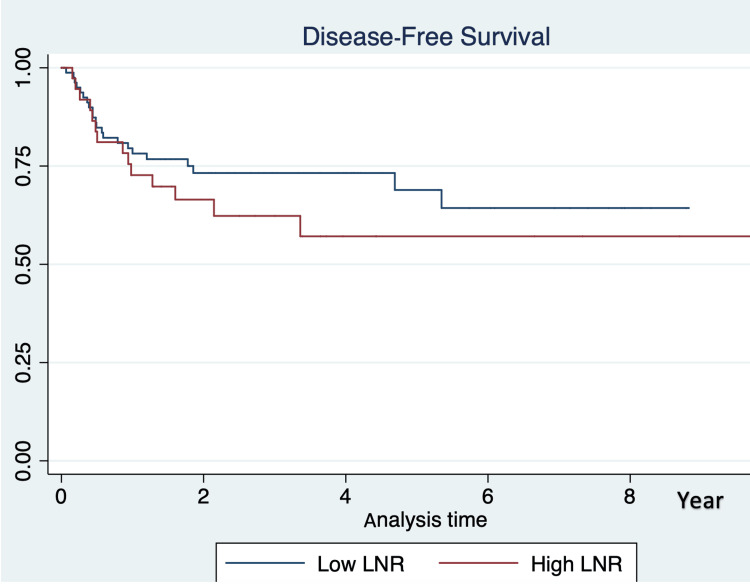
Kaplan–Meier Survival Estimates Showing Four-Year Disease-Free Survival LNR: lymph node ratio

According to the Kaplan-Meier survival estimates for the entire cohort, the four-year DFS was 73% (95% CI: 61% to 82%) for those with a lower LNR and 56% (95% CI: 35% to 72%) for those with a higher LNR, which was statistically significant (Figure 4).

## Discussion

The histopathology reports in our study showed that a median of 38 lymph nodes (mean 43, range 2-228) were dissected during surgery, which is similar to the median number of lymph nodes reported in other studies in the literature [10,11]. After undertaking a thorough review of the literature, we found no reference values for the LNR of head and neck cancer; thus, we characterized the distribution of the individual LNRs in the present patient cohort according to the mean value. The observed mean LNR in the cohort was 0.4, but we could not find a reference in the literature with the same LNR result as ours. Any LNR that fell above 0.04 in our study was considered a high LNR and vice versa.

The observation of our cohort and review of the literature suggest that the prognosis and OS of patients with cancer of the UADT might be related to the LNR. The lower the LNR calculated after neck dissection, the better the prognosis and survival tended to be; i.e., a patient with a mean LNR of lower than 0.4 had a better prognosis, in agreement with multiple studies in the literature [10,11]. In 2010, a German study was conducted that included 384 patients with regionally metastasized oropharyngeal carcinoma who underwent primary surgery between 1980 and 2010 [8]. The five-year disease-specific survival was 73%. An individual LNR peak of 0.1 was closest to the median of 0.0909, and both were set as cutoff values [8]. This means that the LNR is in itself a valuable additional prognostic factor for risk stratification. According to the results of that study, the optimal LNR predictive of survival in oral SCC was expected to be in the range of 0.09 to 0.1 [8]. In a large meta-analysis that included 445 patients between 2013 and 2019, the results showed that a shorter OS, DSS, and DFS were significantly correlated with a higher LNR in a random-effect model. The cutoff values of the eligible studies varied from 0.03 to 0.14, and the lowest significant LNR was 0.044 [12]. Another meta-analysis in Hong Kong used electronic platforms up to January 2018 to evaluate the prognostic effects of LNR and investigate its cutoff value in many centers [9]. Nineteen studies between 2009 and 2017 were included, and the total number of patients was 14,254. Data were categorized into Group A (with pathological nodal disease) and Group B (with and without pathological nodal disease). The high LNR was significantly related to a short OS and DFS in both groups. This finding highlights the fact that LNR can be a good prognostic indicator in oral SCC, suggesting its consideration in future staging systems [9,12].

As surgeons, we cannot control the number of positive lymph nodes that might be present in a neck dissection, but we can maximize the total number of lymph nodes that we excise, regardless of the type of neck dissection. This can be achieved by several means, one of which is to always follow the appropriate steps for neck dissection during surgery, especially in hospital training programs, as the results of training will affect patients and outcomes. All levels of neck dissection should be addressed during surgery in accordance with the type of neck dissection that was planned preoperatively. The better the neck dissection performed, the higher the number of lymph nodes extracted and the lower the LNR, resulting in a better prognosis and OS. Determining a cutoff LNR value would be an important step in the evolution of neck dissection, as it might help determine prognosis and may affect patient survival: From our observations, patients tend to have longer survival periods with lower LNRs (<0.04). Future applications of the LNR might include the use of a precise LNR cutoff value in order to determine the postsurgical treatment plan. This may include whether adjuvant therapy is needed in patients with head and neck cancer with an appropriately performed neck dissection and a low LNR, as well as the possibility of incorporating the LNR in future staging of cancers of the UADT.

Other potential indicators for prognosis discussed in the literature include the yield of lymph nodes during neck dissection or the positive nodal metastasis number. Some studies have been performed to evaluate these measures, with investigators reporting that there is in fact a relationship [8,12] and that these tools can be helpful for prognosis and survival. Ebrahimi et al. [13], suggest that the yield of an appropriate neck dissection is 18 lymph nodes and that excision of over 18 lymph nodes results in a statistically significant better prognosis. When the same lymph node cutoff was applied in other centers, however, the results were close but not statistically significant [14]. From our observations and analysis, the relationship was not quite as strong as with the LNR. Future prospective studies should be performed to further examine this relationship and to determine its statistical significance.

As our study was one of the first of its kind in the Middle East, we faced multiple difficulties that limited the results. First, the initial sample size was not large enough, and because statistical significance was not achieved, we included patients from a second major head and neck center (the National Guard Hospital) to increase the sample size in order to reach statistical significance. Second, some of the patients’ medical records were missing minor information, requiring us to return to the physical files to find the data. Third, given the study design was a retrospective cohort, it led us to a generalized discussion about the LNR and its relationship to the prognosis and survival of patients for all sites of the UADT as a unit. Fourth, the mean follow-up period was only 2.7 years and a longer follow-up time would allow better assessment of long-term survival outcomes. Moreover, the proposed LNR cutoff of 0.04 for prognostic value needs to be validated before it can be widely adopted. Lastly, the study population was predominantly male patients in Saudi Arabia. Findings may not be fully generalizable to female patients or other geographic regions. In summary, the major limitations are the retrospective design, short follow-up duration, and lack of external validation of the proposed prognostic cutoff value.

In the future, expanding the study to a prospective cohort and focusing on each site on its own to better assess the prognostic strength of the LNR rather than generally considering the entire UADT would strengthen the overall conclusions.

## Conclusions

To increase the chance of survival of patients with cancers of the UADT and to improve their prognosis, surgeons should use the best treatment standards available. One of the most important aspects of treatment is appropriate surgery, including that of neck dissection. Appropriate neck dissection requires a maximum number of lymph nodes to be excised to achieve the lowest LNR possible, and hence, better survival and prognosis for patients. Establishment of an LNR should be implemented through further studies and investigation, as it could be of real value for prognosis and survival. Surgeons should always take the LNR into consideration and work precisely, as patients deserve an optimal neck dissection that may affect their prognosis. From our analysis, we consider a mean LNR of less than 0.04 to be low and conclude that a patient with this value has a better chance of survival and a better prognosis. Surgeons should be encouraged to achieve as low an LNR as possible for all patients with cancer of the UADT, so that it can safely be used as a reference in postoperative treatment modalities in anticipation of a good prognosis and longer DFS and OS.
